# Peripheral blood eosinophilia in dogs: Prevalence and associated diseases

**DOI:** 10.1002/vms3.832

**Published:** 2022-06-02

**Authors:** Abigail Guija‐de‐Arespacochaga, Loïc Kremer, Frank Künzel, Ilse Schwendenwein

**Affiliations:** ^1^ Department of Pathobiology Clinical Pathology Platform, University of Veterinary Medicine Vienna Vienna Austria; ^2^ Korneuburg AniCura Tierklinik Korneuburg Austria; ^3^ Department for Companion Animals and Horses Small Animal Clinic Internal Medicine University of Veterinary Medicine Vienna Vienna Austria

**Keywords:** hypereosinophilia, hypersensitivity, neoplasia, parasites

## Abstract

**Background:**

Canine eosinophilia has not been evaluated over the last two decades. As in human local differences, changes in the prevalence and associated diseases over time can be expected.

**Objective:**

This study aims to determine the prevalence and causes of marked blood eosinophilia in dogs.

**Methods:**

Retrospective study. A total of 317 clinical histories of dogs with an eosinophil concentration > 1.5 × 10^9^/L (marked eosinophilia) between 2013 and 2017 were evaluated. Patients were allocated to 10 groups according to their major clinical findings.

**Results:**

Eosinophilia was present in 1,592 of 10,829 dogs (14.7%); it was mild (0.8–1.49 × 10^9^/L) in 78.4%, moderate (1.5 – 4.9 × 10^9^/L) in 20.5% and severe (> 5 × 10^9^/L) in 1.1% of cases. Rottweilers were overrepresented (16.1%). Of 317 cases with marked eosinophilia, 19.6% had neoplasia, 19.1% gastrointestinal disorders, 13.6% health check, 10.4% endoparasites, 6% respiratory, 5.4% neurologic, 5.4% dermatologic, 4.8% urogenital, 3.2% endocrine disorders and 12.6% miscellaneous. Lymphomas (29%) and mast cell tumours (12.9%) were the most frequent tumours in the neoplasia group. A total of 72.6% of tumour‐bearing dogs were older than 8 years, while 63.6% of dogs had endoparasites, and 86% of apparently healthy dogs were younger than 5 years. Eosinophilia was significantly higher in patients with respiratory disorders (*p* < 0.0146). Leukocytosis was found in 50.2% of cases.

**Conclusion:**

Malignancy was the most common cause of marked blood eosinophilia in older dogs and endoparasitism in younger dogs. Eosinophilia was common in apparently healthy young dogs and may be related to undiagnosed parasitic infestations.

## Graphical abstract

We determine the prevalence and causes of marked blood eosinophilia in dogs. We found local differences compared to previous studies, being malignancy a common cause in adult dogs while parasitic infestation remains the most frequent cause in young animals.

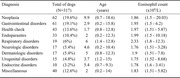

## 1 INTRODUCTION

Peripheral blood eosinophilia is related to a wide variety of causes, ranging from hypersensitivity and parasitic infestations to neoplasia. As in people, it seems that local differences in the incidence also exist in animals (Lilliehöök et al., 2000; O'Connell & Nutman, 2015). While in resource‐limited countries, parasitic infestations represent the most common cause of eosinophilia in people, hypersensitivity reactions should be considered a primary differential diagnosis in developed countries. Likewise, it can be expected that climatic changes affect the distribution of intermediate hosts necessary for the replication of helminths or the spread of infections transmitted by ticks, which finally leads to a change in the distribution of parasites that could be reflected in a local change in causes of eosinophilia (Skuce et al., 2013). Even if the causes of eosinophilia are well known, studies in local dog populations are scarce, and recent data are missing—the last one was published about two decades ago (Lilliehöök et al., 2000). Moreover, changes in the prevalence of eosinophilia and associated diseases in Central Europe can be expected as a result of changes within the dog population (e.g., spectrum of breeds, age) or the increase in certain immunologic or neoplastic diseases. The latter is associated with increased life expectancy and better veterinary care for geriatric pet animals. The aim of this retrospective study was to determine the prevalence, grade and associated diseases in dogs with marked peripheral blood eosinophilia presented to the Small Animal Clinic of the University of Veterinary Medicine, Vienna over a 4‐year period.

## 2 MATERIALS AND METHODS

A retrospective study was carried out on medical records of dogs with peripheral eosinophilia presented to the Small Animal Clinic of the University of Veterinary Medicine, Vienna between January 2013 and December 2017. Eosinophilia was defined as an eosinophil count of ≥ 0.8 × 10^9^/L (upper cut‐off applied in the laboratory) and was classified as mild < 1.5 × 10^9^/L, moderate ≥ 1.5 to < 5.0 × 10^9^/L and severe ≥ 5 × 10^9^/L (Boyer, 2016). Marked eosinophilia was defined by eosinophil counts ≥ 1.5 × 10^9^/L (Valent et al., 2012). Only patients with marked blood eosinophilia, a complete clinical history and a definitive diagnosis were included in the study. Medical records of these dogs were reviewed for signalment (breed, gender and age), the results of diagnostic tests and final diagnosis.

An automatised complete blood cell count (CBC) was performed by the ADVIA 2120i (Siemens Healthcare Diagnostics GmbH, Austria). Blood smears from all patients were prepared and stained by an automated stainer using a modified Wright stain (Hematek and Hematek Stain Pack, Modified Wright; Siemens Healthcare Diagnostics GmbH). According to our standard operating procedures, haematologic evaluations are performed by a senior technician according to the following guidelines for the leukogram. Numerical changes in total leukocyte counts, neutrophils and lymphocytes exceeding 25% of the upper or lower cut off for each cell population must be inspected by microscopy regardless of the scatterplots. Any monocyte and eosinophil count above 1 × 10^9^/L warrants microscopic slide inspections. In the case of misclassification of cells by the instrument, such as counting eosinophils as monocytes due to low peroxidase activity, a microscopic differential is performed. Additionally, scatterplots with indistinct separation of cell populations warrant microscopic inspection.

Only the results of the first consultation were included in the study unless different causes for eosinophilia were established for the same patient. Based on clinical data, patients were grouped into 10 categories referring to the most heavily affected organ system or aetiology: gastrointestinal, dermatologic, neurologic, respiratory, urogenital as well as neoplastic, endocrine disorders, endoparasites, miscellaneous and health check. The review of the medical records allowed further classification based on the final diagnosis.

According to their age, patients were divided into four categories: young (< 1 year old), young adult (1–4.9 years old), adult (5–7.9 years old) and senior (≥ 8 years old).

Statistical analysis was performed with the statistics add‐in software for Microsoft Excel Analyse‐it (Analyse‐it, version 5.65). Visual inspection of dot plots of eosinophil counts in the different groups was performed. Numerical variables were assessed for normality using the Shapiro–Wilk test. Data were not normally distributed, and comparisons between groups were conducted using the Tukey–Kramer test. Values of *p *< 0.05 were considered statistically significant.

## 3 RESULTS

During the study period, 21,698 CBCs were performed on 10,829 dogs at the Central Laboratory of the University of Veterinary Medicine, Vienna. Eosinophil counts above 0.8 × 10^9^/L were found in 1,592 samples (14.7%). When stratified by severity, eosinophilia was classified as mild in 1,248 (78.4%), moderate in 326 (20.5%) and severe in 18 (1.1%) samples. The results of the study focus on the patients with marked eosinophilia for whom the medical records have been reviewed. A total of 317 clinical histories from 314 dogs (one dog was included two times and another three times because of different final diagnoses during the study period) were included in this study. The mean age was 5.3 years, with a total of 47 (15%) young, 126 (40.1%) young adult, 49 (15.6%) adult and 92 (29.3%) senior dogs. The sex distribution was 52.6% females (25.2% spayed) and 47.5% males (18.5% neutered). Out of 314 dogs, 110 (35%) were mixed‐breed dogs, and the remaining dogs represented 85 breeds, including Rottweiler (18; 5.7%), German shepherd dog (13; 4.1%), Labrador (12; 3.8%) and Chihuahua (11; 3.5%); other breeds were represented with less than 3% of the population. Rottweilers were represented by 112 individuals in the entire cohort searched for eosinophilia. Eighteen out of these 112 (16.1%) individuals showed marked eosinophilia. Other overrepresented breeds among dogs with marked eosinophilia include German shepherd dogs 6.1% (13/213; median 0.49 × 10^9^/L), mixed‐breed dogs 4.2% (113/2705; 0.38 × 10^9^/L), Labradors 3.6% (12/333; 0.42 × 10^9^/L) and Chihuahuas 2.7% (11/408; 0.28 × 10^9^/L; Table 1).

**TABLE 1:** Dog breeds with a high prevalence (more than 10 dogs) of marked eosinophilia (≥1.5 × 10^9^/L, *n* = 314) in relation to the total of canine complete blood cells counts for the same breeds during the study period

**Breed**	**Number of dogs per breed within the total sample**	**Dogs with marked eosinophilia**	**Eosinophil count median (x10^9^/L)**
Rottweiler	112	18 (16.1%)	0.77
German shepherd dog	213	13 (6.1%)	0.49
Mixed breed	2705	110 (4.2%)	0.38
Labrador retriever	333	12 (3.6%)	0.42
Chihuahua	408	11 (2.7%)	0.28

*Note*: The eosinophil count in Rottweilers was significantly higher than that in the other overrepresented breeds (*p* < 0.0003).

Dogs with neoplasia (*n* = 62; 19.6%) formed the largest group, followed by 61 (19.1%) dogs with gastrointestinal disorders, 33 (10.4%) dogs with endoparasitoses, 19 (6%) with respiratory and 17 (5.4%) with neurologic disorders, 17 (5.4%) with dermatologic disorders, 15 (4.8%) with urogenital disorders and 10 (3.2%) with endocrine disorders. In 43 (13.6%) dogs, eosinophilia was an incidental finding during a health check, and 40 (12.6%) dogs had to be allocated to the miscellaneous group (Table 2).

**TABLE 2:** Allocation of 317 dogs with marked eosinophilia (≥ 1.5 × 10^9^/L) into diagnostic groups

	**Total of dogs**	**Age**	**Eosinophil count**
**Diagnosis**	**(*N* = 317)**	**(years)**	**(x10^9^/L)**
Neoplasia	62 (19.6%)	9.9 (0.7–18.6)	1.86 (1.5–20.03)
Gastrointestinal disorders	61 (19.1%)	2.9 (0.2–15.8)	1.95 (1.5–6.2)
Health check	43 (13.6%)	1.7 (0.8–12.8)	1.97 (1.51–5.87)
Endoparasites	33 (10.4%)	2 (0.2–12.3)	1.99 (1.5–10.18)
Respiratory disorders	19 (6%)	6 (1.4–12.8)	2.33 (1.8–12.3)
Neurologic disorders	17 (5.4%)	4.6 (0.2–10.4)	1.76 (1.51–3.28)
Dermatologic disorders	17 (5.4%)	5 (0.8–12.3)	1.9 (1.51–7.8)
Urogenital disorders	15 (4.8%)	3.7 (1.2–15)	1.75 (1.52–8.68)
Endocrine disorders	10 (3.2%)	5.4 (0.7–9.3)	1.78 (1.6–3.41)
Miscellaneous	40 (12.6%)	2 (0.2–14)	1.83 (1.51–5.82)

*Note*: Age and eosinophil count are reported as median and range (in brackets).

**FIGURE 1:**
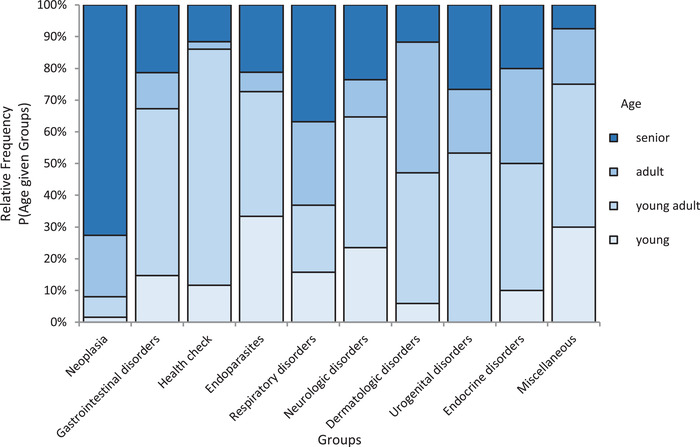
Age distribution (as a percentage) of dogs with marked eosinophilia (≥ 1.5 × 10 cells/L; Lyles et al., 2009) within the diagnostic categories: young (< 1‐year old), young adult (1–4.9 years old), adult (5–7.9 years old) and senior (≥ 8 years old)

Among dogs with neoplasia, 18 had lymphoma (29%), and eight had a mast cell tumour (12.9%), followed by transitional cell carcinomas, hemangiosarcomas and soft tissue sarcomas, each represented by three patients. Other tumour types only appeared once or twice. Immunophenotype was available in 13 dogs with lymphoma; eight dogs presented a B‐cell lymphoma, and five dogs presented a T‐cell lymphoma. In five dogs, the immunophenotype was not determined. Eosinophilia was moderate in all but one dog in this group (median 1.86, range 1.5–20.03 × 10^9^/L). Senior dogs were overrepresented in this group (Figure 1); forty‐five dogs (72.6%) with neoplasia were older than 8 years (median 9.9, range 0.7–18.6 years); only one young dog was in this category. This 7‐month‐old dog was diagnosed with acute myeloid leukaemia and showed the highest eosinophil and leukocyte counts in the present study (20.03 × 10^9^/L and 143.1 × 10^9^/L, respectively).

Sixty‐one dogs had gastrointestinal disorders, of which 24 (39.3%) had gastroenteritis (18 acute and six chronic). Of the remaining 37 dogs, eight had acute haemorrhagic diarrhoea syndrome, seven had inflammatory bowel disease, five had parvovirosis, four had pancreatitis and food intolerance and three had gastrointestinal bleeding, while stomatitis, esophagitis and foreign bodies were present in two dogs each. Forty‐one dogs (67%) in this group were younger than 5 years (median 2.9, range 0.2–15.8 years). Eosinophilia was moderate in all but one dog in this group (median 1.95; range 1.5–6.2 × 10^9^/L). Only one 2‐month‐old dog with severe acute gastroenteritis showed severe eosinophilia. Additionally, this dog showed a leukaemoid reaction (103 × 10^9^/L), and endoparasites and parvovirosis tested negative several times.

Forty‐three (13.6%) dogs were presented for health checks. These dogs were apparently healthy and included blood donors or dogs that were clinically evaluated before elective surgery (castration, minor surgical procedures). Thirty‐seven (86%) dogs in this group were younger than 5 years (five young and 32 young adults; median 1.7, range 0.8–12.8 years). Severe eosinophilia was found in only one dog (median 1.97; range 1.51–5.87 × 10^9^/L). This dog, a Greyhound from a Spanish shelter, was presented for entropium surgery. This patient was dewormed and tested negative for leishmaniosis.

Thirty‐three dogs (10.4%) tested positive for endoparasites. Parasitism consisted of four dogs with *Trichuris* spp., *Giardia* spp., *Cystoisospora*, three with *Dirofilaria immitis* and *repens, Toxocara canis*, and two with *Sarcocystis* spp. and *Toxoplasma gondii*, *Crenosoma* spp. One dog with *Babesia canis*, one with *Leishmania infantum* and two with *Anaplasma phagocytophilum* were also included in this group. A total of 63.6% of dogs were younger than 5 years (median 2, range 0.2–12.3 years). The highest eosinophil concentration was found in a puppy with a severe infestation with *Trichuris* spp. and *Toxocara* spp. (median 1.99, range 1.5–10.18 × 10^9^/L).

**FIGURE 2:**
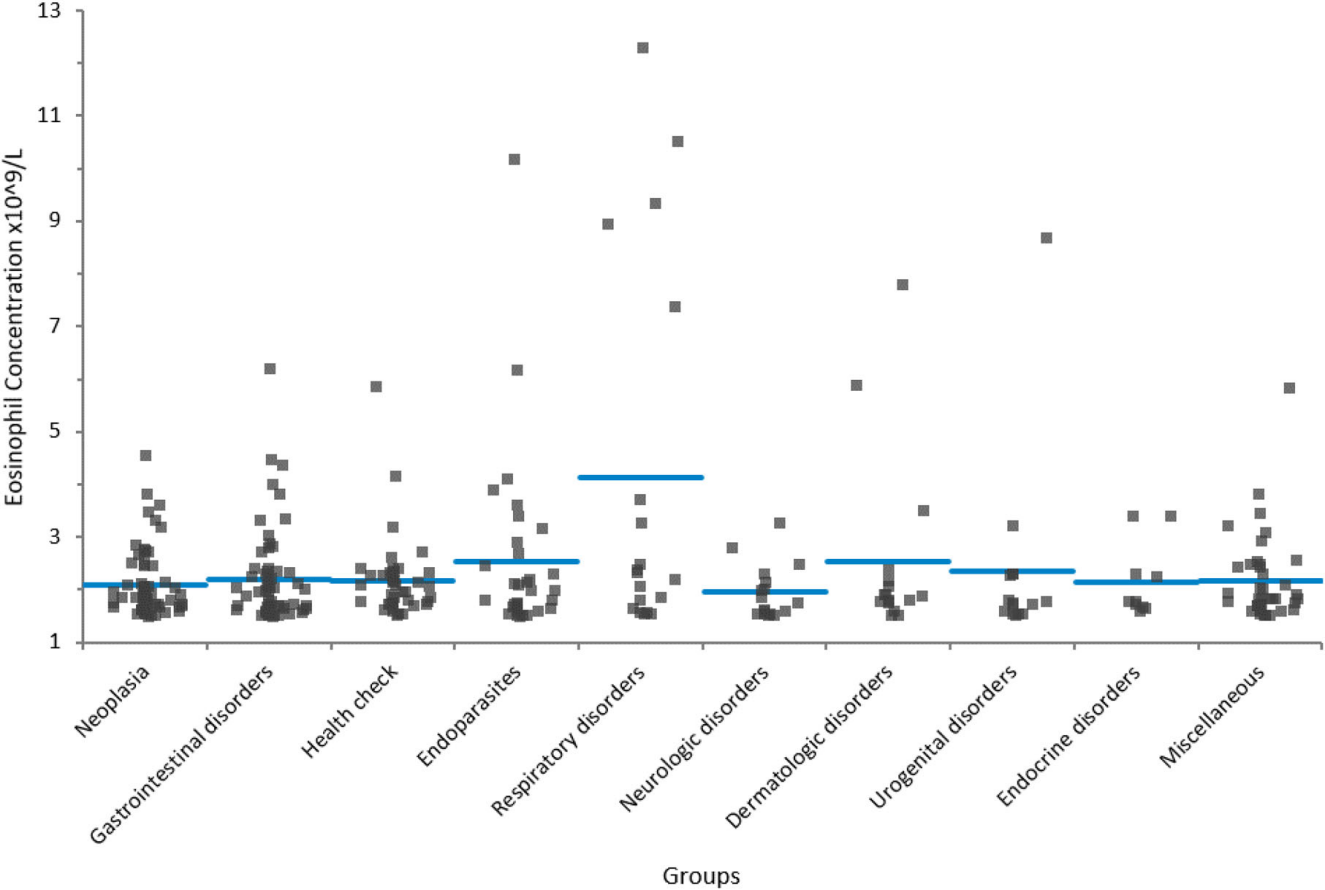
Comparison of eosinophil concentration among groups in dogs (*n *= 317) with marked eosinophilia (≥ 1.5 × 10^9^/L). Eosinophilia was significantly higher in dogs with respiratory disorders than in other groups (*p* < 0.0146). The horizontal line represents the median. An outlier in the neoplasia group with 20.03 × 10 cells/L (Lyles et al., 2009) was removed to improve display

Respiratory disorders were diagnosed in 19 (6%) dogs. The diagnosis included 14 animals with bronchopneumonia, of which seven patients had severe eosinophilic infiltration within the bronchoalveolar lavage fluid (BALF). Of the remaining five dogs in this group, two dogs had a pyothorax, while pneumothorax, lung contusion after a car accident and chronic rhinitis were present each in one patient. Eosinophilia (median 2.33, range 1.8–12.3 × 10^9^/L) was significantly higher in patients with respiratory disorders than in other groups (*p* < 0.0146) (Figure 2). Severe eosinophilia was present in 26.3% (five) of the dogs. In all of these dogs, the final diagnosis revealed severe eosinophilic bronchopneumonia. Two patients in whom endoparasites were confirmed by coproscopy or other means were included in the endoparasite group.

Seventeen (54%) dogs presented a dermatologic disorder, which included seven dogs with dermatitis. Two of these dogs had a diagnosis of eosinophilic dermatitis, with severe blood eosinophilia in one dog. Other diagnoses included atopic dermatitis and otitis externa represented by four dogs each, as well as two with pemphigus foliaceus and one with a flea infestation. Eosinophilia (median 1.9, range 1.51–7.8 × 10^9^/L) was severe in only two dogs. Young adult and adult dogs represent 82.4% of all dogs, each with half (median 5, range 0.8–12.3 years).

Neurologic disorders were the final diagnosis in 17 dogs (5.4%); eight of these had primary epilepsy, four physical spinal cord damage and three hydrocephalus, while one had meningitis and one an autoimmune disease. A cerebrospinal fluid analysis was conducted in five out of 17 patients, of whom only one dog with granulomatous meningoencephalitis showed lymphocytic pleocytosis. Eosinophilia (median 1.8, range 1.5–3.3 × 10^9^/L) was moderate in all but one patient. Seven dogs (42.7%) were young adults (median 4.6, range 0.2–10.4 years).

Sixteen (5.1%) dogs had urogenital disorders; most of those were related to a bacterial infection: pyometra (six), urinary tract (three), as well as mastitis and epididymitis. The remaining dogs (four) were all females with ovarial‐uterine dysfunctions. Eosinophilia (median 1.75, range 1.52–8.68 × 10^9^/L) was severe in only one dog diagnosed with pyometra. Eight (53.3%) patients were young adults (median 3.7, range 1.2–14.9 years).

Nine out of 10 dogs with endocrine disorders had hypoadrenocorticism, while diabetes mellitus was diagnosed in only one dog originating from a shelter. Eosinophilia (median 1.78, range 1.6–3.41 × 10^9^/L) was moderate in all patients. Most of the dogs were young and young adult dogs, each with 40% (median 5.4, range 0.7–9.3 years).

The remaining 40 (12.6%) dogs were included in the miscellaneous category and represented several diseases of the musculoskeletal system (*n* = 11), cardiovascular (*n* = 8) and ophthalmologic (*n* = 6), tooth fractures (*n* = 4), intoxications (*n* = 2), post‐surgical complications (*n* = 2), unexplained lymphadenomegaly (*n* = 2) and eosinophilic lymphadenitis, among others. Eosinophil counts (median 1.83, range 1.51–5.82 × 10^9^/L) were the highest in one dog included in this group with a suspected hypereosinophilic syndrome (HES).

## 4 DISCUSSION

The aim of the present study was to determine the prevalence of blood eosinophilia in dogs and to determine the cause of marked eosinophilia in the Vienna City area, Austria. Causes of peripheral blood eosinophilia are well‐established, but as in humans, regional differences should be expected (Barrett et al., 2017). Only one paper has reviewed the causes of blood eosinophilia in dogs within the last 20 years (Lilliehöök et al., 2000). In that study, the prevalence for eosinophilia was 10%, given a cut‐off of 1.25 × 10^9^/L. When we consider our laboratory's reference value of 0.8 × 10^9^/L, the prevalence of eosinophilia in the present study was 14.7% and 5.2% when using the same cut‐off as the previous Swedish study and 3.17% for marked eosinophilia (≥ 1.5 × 10^9^/L). As in Sweden, we also found differences among breeds; Rottweilers showed more frequent eosinophilia and exhibited significantly higher eosinophil counts, compared with other breeds (Lilliehöök et al., 2000). This could be related to a predisposition of Rottweilers for eosinophilic disorders such as eosinophilic gastrointestinal (GI) diseases and meningoencephalitis as well as the rarely diagnosed HES (Bennett et al., 1997; Lyles et al., 2009; Sykes et al., 2001).

Interestingly, a high percentage of patients with marked eosinophilia suffered from neoplastic disease. Unsurprisingly, this was especially true in older dogs. With a total of 62 out of 317 dogs (19.6%), these results differ from the study in Sweden, in which only four dogs out of 105 (3.8%) presented with neoplasia. However, an active oncologic referral centre at the Small Animal Clinic might have introduced an observation bias in the investigated study cohort. While primary (clonal) eosinophilia as a result of overproduction of eosinophils in myeloid neoplasms by abnormal progenitor cells is only rarely reported, paraneoplastic eosinophilia has been associated with haematologic malignancies such as lymphomas and several solid tumours (Gotlib, 2017). In the present study, lymphomas represent 29% by far the largest group among neoplasia‐associated eosinophilia. In contrast to previous studies, in our cohort, B‐cell lymphomas were more frequently associated with eosinophilia (eight out of 13) than T‐cell lymphomas (Marchetti et al., 2005; Ozaki et al., 2006). However, B‐cell lymphomas are only occasionally reported to be associated with eosinophilia (Tomiyasu et al., 2010). Mast cell tumours were the second most frequently diagnosed neoplasia, while eosinophilic tissue infiltration is known to be a common finding in this type of tumour, and peripheral eosinophilia is only rarely documented (O'Keefe et al., 1987; Skor et al., 2017; Takahashi et al., 2000; Tomiyasu et al., 2010). So far, only one report has described a case of a dog with severe eosinophilia (24 × 10^9^/L) having a cutaneous low‐grade mast cell tumour. Eosinophils returned to normal concentrations after starting therapy with prednisone, increasing again after recurrence and lymph node metastasis (Musser et al., 2018). Nevertheless, in a smaller number of dogs, we found that eosinophilia was present in a wide variety of other neoplasms. However, the association with non‐haematologic tumours, especially carcinomas, is well known (Losco, 1986; Samoszuk, 1997). The role of eosinophils in neoplasia is not clear; possible mechanisms are related to remodelling of the connective tissue after tissue damage by the growing tumour, but cytotoxic effects of eosinophils on tumour cells have also been suggested (Davis & Rothenberg, 2014). The release of protein material through necrotic changes as well as cytokines such as interleukin‐5 (interleukin [IL]‐5), IL‐3 and eotaxin‐1 produced by tumour or host inflammatory cells attract eosinophils to neoplastic tissue. On the other hand, blood eosinophilia results from the stimulation of the bone marrow by colony‐stimulating factors produced by some specific neoplastic cells (Samoszuk, 1997). The significance of eosinophilia in tumour behaviour is not clear, but blood eosinophilia seems to be related to widespread metastases and poor prognosis. The fact that eosinophils return to normal values after treatment and increase again with recurrence or metastasis in patients with neoplasia can help to predict the course of the disease (Musser et al., 2018).

GI disorders (19.1%) represented the second most common cause of eosinophilia in the dogs of the present study, comparable to the Swedish survey, where 25% of cases were in this group. Even if peripheral eosinophilia is an inconstant finding in patients with GI disorders (Talley et al., 1990), it has been related to acute and chronic GI diseases (Lilliehöök et al., 2000; Mehta & Furuta, 2015), and less frequently with other inflammatory conditions such as pancreatitis, especially when severe tissue injury to adjacent organs occurs (Tokoo et al., 1992). Eosinophils seem to play an important role in the GI tract not only in the sense of defence against parasites but also in the case of bacterial and viral infections, preventing uncontrolled bacterial invasion after damage to intestinal epithelial cells, as seen in dogs with parvovirosis (Goddard & Leisewitz, 2010; Yousefi et al., 2008).

In contrast to the most recent study in Sweden (Lilliehöök et al., 2000), where only one dog with a hookworm infestation was documented, endoparasitism was more frequently associated with eosinophilia in the present study (10.4%). Helminthiases have a reputation to produce a marked peripheral eosinophilia. The degree and duration of eosinophilia are determined by the development, migration and distribution of the parasite and the immune response of the host, being high during tissue migration and lower with intraluminal parasites or in those that produce cysts unless these become disrupted (Nutman, 2007). Other endoparasites, such as *Giardia* spp. and intestinal coccidial infections have been related to a lesser extent to eosinophilic tissue infiltration (Aloisio et al., 2006) and peripheral blood eosinophilia (Center et al., 1990). Some authors suggest that *Giardia* spp. may produce some allergen, which could reach a deeper layer of intestinal mucosa during infection, resulting in eosinophilia (Dos Santos & Vituri, 1996).

The association between eosinophilia and parasite infestation is hampered by the fact that it can take months for eosinophils to return to normal values after antiparasitic treatment (Leder & Weller, 2000). On the other hand, eosinophilia may be present in parasitised dogs with a negative coproscopic result. Nevertheless, the prevalence of intestinal parasitosis in dogs in developed European countries reaches values between 30% and 50% for pets living in cities and metropolitan areas, respectively, being most common in dogs younger than 1 year (Barutzki & Schaper, 2011; Zanzani et al., 2014) This fact might explain the high number of patients (*n* = 43) with eosinophilia in the health check group of the present study. Similar to the endoparasite group, the majority of these dogs were younger than 5 years, and even if no additional diagnostic tests were performed, antiparasitic treatment against intestinal parasites was applied. However, the parasitic infection cannot be excluded in dogs of this group.

While respiratory disorders reached 17% of the cases with eosinophilia in the study of Lilienhöök, only 6% of cases with eosinophilia were associated with respiratory disease in the present study. A common feature of both studies was the grade of eosinophilia, which was higher, compared to the other groups. Pulmonary eosinophilia is characterised by an infiltration of the lung tissue, with values greater than 15% eosinophils in the BALF indicative of pathologic eosinophilic infiltrate (Johnson et al., 2019). Severe eosinophilic lung infiltration was confirmed by BALF analysis in half of the patients with respiratory disease. Pulmonary infiltrates with eosinophilia are a diverse group of lung diseases with a wide range of presentations characterised by lung tissue eosinophilia with or without peripheral eosinophilia (Clercx et al., 2000). In a recent study, eosinophilic lung disease was classified into three categories, eosinophilic bronchitis (EB), granuloma (EG) and bronchopenumopathy, based on radiography, bronchoscopy, BALF and haematologic findings; in patients with EB, peripheral eosinophilia was rare, percentages of eosinophils in BAL fluid were low, and only minimal bronchoscopic changes were present, while changes, including bronchiectasis, were more severe in the last two groups, suggesting more marked airway inflammation with consecutive tissue damage. Recognising peripheral severe eosinophilia can help in the classification and in determining the prognosis of these patients, reported as guarded in patients with EG (Johnson et al., 2019). Determining the cause of eosinophilia in these patients may be challenging since many different factors may lead to pulmonary hypersensitivity, such as exposure to certain microorganisms (parasites, fungal and bacterial infections) and chemical substances (toxic products, medications), among others. In many cases, the cause cannot be elucidated, but knowing the prevalence of some infectious diseases, such as parasite infections, is essential for diagnostic workup. This is of great importance in the case of helminthiasis, for which prevalence rates might differ significantly among countries (Giannelli et al., 2017). In some cases, diagnosis can be easily overlooked, since larvae are rarely present in fecal or transtracheal wash samples, and antiparasitic therapy should be considered in these patients even when faecal examinations are negative (Shaw et al., 1996). Negative results are expected in the early phases of infection and remain negative for up to 8 weeks after the onset of pulmonary signs (Campos, 2009).

Similar to endoparasites, differences were found in dogs with skin disorders when compared with the study in Sweden (Lilliehöök et al., 2000), where 7% of dogs in the Swedish study had a *Sarcoptes scabiei* infection, and none was detected in our study. In that sense, flea allergy dermatitis was the most common cause of peripheral eosinophilia in cats (20%) in another study (Center et al., 1990), compared to only two dogs (0.63%) in the present study. Peripheral eosinophilia is a common but inconstant finding in eosinophilic dermatoses, even in patients with severe eosinophilic infiltration (Mauldin et al., 2006), and as already reported, it was related, in addition to parasitic infestations, to allergic reactions and nonspecific dermatopathies, including bacterial dermatitis and autoimmune disorders, such as pemphigus foliaceus (Center et al., 1990; Lilliehöök et al., 2000).

Hypoadrenocorticism was diagnosed in all but one dog with an endocrine disorder. Eosinophilia is one of the hallmarks of these patients, being present in 20% of cases, and hypoadrenocorticism should always be considered in chronically ill patients with eosinophilia and a concurrent sodium:potassium ratio lower than 27:1 (Peterson et al., 1996). Additional calculation of neutrophil to eosinophil as well as to lymphocyte ratio might help in the diagnosis of hypoadrenocorticism when adrenocorticotropic hormone (ACTH) stimulation test or measurement of ACTH concentrations are not available since these differ from dogs with other diseases (Zeugswetter & Schwendenwein, 2014).

Hypereosinophilia refers to marked blood eosinophilia (≥1.5 × 10^9^/L) for at least 6 months with or without tissue eosinophilia and is further classified as HES when damage to end organs is present together with no apparent aetiology or evidence of clonality (Davis & Rothenberg, 2014). This is a very rare disease in humans, in which diagnosis is usually made per exclusion. In many cases, HES has been re‐classified as primary clonal eosinophilia after confirmation of cell clonality (Brito‐Babapulle, 2003). In veterinary medicine, a tentative diagnosis of HES is commonly made if no other relevant causes are detected after deworming of patients with chronic hypereosinophilia.

In conclusion, as reported in humans, we found a clear difference in the distribution of causes of marked blood eosinophilia in dogs when compared to previous studies, and, surprisingly, the most common cause of marked eosinophilia in the current study was malignancy. This may be due to a higher incidence of diseases such as cancer, the age of the population under investigation, an observational bias due to the local oncologic referral centre and absence of infectious diseases such as heartworms that are more common in southern countries. However, the high number of apparently healthy young patients with eosinophilia in our study raises suspicion of undetected parasitic infestation. Therefore, parasitic infestation should be considered in puppies and young adults with unknown eosinophilia, while neoplasia should become part of differential diagnoses in older patients.

## CONFLICT OF INTEREST

The authors declare no conflict of interest.

## ETHICS STATEMENT

This study is a retrospective study and did not require Ethics Committee approval.

## AUTHOR CONTRIBUTIONS

*Design, data analysis, supervision of project, writing–original draft and review and editing*: Guija‐de‐Arespacochaga. *Data collection, data analysis, writing–original draft*: Kremer. *Writing–review & editing*: Künzel. *Design, data analysis and processing, writing: review and editing*: Schwendenwein.

### PEER REVIEW

The peer review history for this article is available at https://publons.com/publon/10.1002/vms3.832

## Data Availability

The data that support the findings of this study are available on request from the corresponding author.
